# Does the health system model shape prevention? Evidence from 22 OECD countries (2004–2023)

**DOI:** 10.3389/fpubh.2026.1836855

**Published:** 2026-06-08

**Authors:** Pietro Marraffa, Lorenzo Marega, Gianfranco Politano, Maria Michela Gianino

**Affiliations:** 1 Department of Sciences of Public Health and Paediatrics, University of Turin, Turin, Italy; 2 Department of Control and Computer Engineering, Polytechnic of Turin, Turin, Italy

**Keywords:** healthcare systems, OECD, prevention, preventive care expenditure, time trend analysis

## Abstract

**Introduction:**

Against a background of population ageing, rising healthcare costs, and growing public-health challenges, this study analysed trends in the share of total health expenditure allocated to preventive care from 2004 to 2023 in 22 European countries, examining whether specific health systems are associated with different relative budgetary prioritisation of preventive care. Although there are few studies investigating this issue adopting the standard tripartite classification, to our knowledge, this is the first study to explore the topic using the latest classification of healthcare systems proposed by Böhm.

**Methods:**

We conducted a time-trend analysis using secondary data from 22 European OECD countries over a twenty-year period (2004–2023); in addition, a hierarchical semi-log polynomial mixed-effects regression analysis has been performed, including annual country-level percentage of total health expenditure allocated to preventive care in association with the three structural dimensions – regulation, financing and provision — according to Böhm's classification as explanatory variables.

**Results:**

Our results indicate that, in terms of compound annual rate, most countries exhibited an increase in the preventive-care expenditure share (between 0.2% and 3.7%), while seven countries showed a decrease (between −6.3% and −0.2%) during the considered period. The regression analysis shows that the trend of the preventive-care expenditure share did not differ in two of the three dimensions under study: financing and provision. In contrast, in countries with societal regulation, the curvilinear trend was more pronounced than in countries with statal regulation (*b* = 0.0035; 95% CI = 0.0013, 0.0057).

**Discussion:**

In conclusion, we found limited evidence that health-system type was associated with the relative share of health expenditure allocated to preventive care in the countries analysed; these findings suggest that the relative budgetary prioritisation of preventive care may be shaped less by formal health-system architecture than by contextual factors, including political priorities, public-health strategies, and major external shocks.

## Highlights

- **What is already known on this topic?**
  *Preventive care represents a relatively small share of total health expenditure in most OECD countries, despite its recognized importance in addressing public health issues. Previous studies attempted to explore cross-country differences in preventive spending and the potential role of healthcare system organization, often using traditional classifications (e.g., Beveridge or Bismarck). However, evidence remains limited and no studies have examined long-term trends using current multidimensional classifications of healthcare systems*.
- **What does this study add?**
  *By analyzing trends in the share of total health expenditure allocated to preventive care over a twenty-year period across 22 European OECD, our study found limited evidence that trends in the preventive-care expenditure share differed systematically across broad health-system structural dimensions. Among the analyzed dimensions, only the regulation showed a more pronounced curvilinear trend in countries with societal regulation*.
- **How this study might affect research, practice or policy?**
  *The findings suggest that the relative priority assigned to preventive care within health expenditure may depend more on contextual factors such as political priorities and public health strategies rather than structural characteristics of healthcare systems. Policymakers should therefore promote prevention through targeted policy commitment instead of relying on health system design alone*.

## Introduction

In an era of aging populations, rising healthcare costs, and growing global health challenges, the need for health promotion and disease prevention has become increasingly prominent, with preventive healthcare identified as a key health policy priority by the European Commission for 2024–2029 (1).

To provide some context, it should be noted that, throughout this paper, the term ‘prevention' will refer to any measure that aims to avoid or reduce the number or the severity of injuries and diseases, their sequelae and complications; furthermore, by the terms ‘preventive care expenditure' or ‘public health spending', the authors refer to the amount of financial resources invested in information, education and advice programmes, immunization programmes, early disease diagnosis programmes, health monitoring programmes, epidemiological surveillance and risk and disease control programmes, as well as in disaster preparedness and emergency response programmes (2).

While there is a consensus on the prevention of the diseases as the most valuable tool in order to reduce the burden of disease and associated risk factors (3), there is no homogeneity in the behavior regarding the expenditures on prevention by different countries, besides some governments invest more than others in public health services, as is suggested by the literature on health care systems (4–7). However, the few studies that investigated the relationship between preventive care expenditures and the type of healthcare system found that the latter is a determinant of preventive care expenditure patterns, adopting the classic ideal-typical distinction between national health insurance, societal health insurance and the private healthcare system (4, 5). The breakdown of healthcare systems based on these three ideal types can be considered the standard tripartite classification (8) and, to our knowledge, the only exception consisted in a study that has adopted a different classification of healthcare systems conducted by Trein (5). The study hypothesized the relationship between preventive and curative fields depends on the healthcare system in a country, that is, the distinct forms of governance in three dimensions: regulation, financing, and provision of healthcare (9).

Over the past few decades, healthcare expenditures have become a key indicator of governments' commitment to the wellbeing of their populations. Yet, performances on preventive care expenditures is a field that needs to be studied in greater detail. The literature documented a lack of funding for preventive care (4) and cuts to public health budgets, especially, after the economic crises (10) but very little studies on the trend of preventive care spending over time has been conducted (4, 10, 11): in fact, while previous studies have described preventive care expenditure levels across countries and others have analyzed overall health spending trends, there is a lack of longitudinal, comparative analyses specifically focused on preventive care expenditure across European countries and stratified by healthcare system characteristics (12, 13). Recent comparative studies on OECD countries have increasingly emphasized the importance of analyzing health systems through multidimensional frameworks, incorporating elements such as system performance, resilience, governance and prevention strategies, alongside their orientation toward primary care, prevention, and performance management. For instance, Reibling et al. (14) propose an extended typology of OECD healthcare systems that integrates institutional characteristics (e.g., financing, access regulation) with performance-related dimensions, including prevention and quality of care. Their findings show that healthcare systems can be grouped into distinct clusters based on structural and functional features, with significant cross-country variation in terms of prevention efforts and health outcomes. This highlights the relevance of considering system typologies when analyzing differences in health expenditure patterns. In addition, in a recent comparative analysis of health policies, Jialu Song et al. (15) examined changes in the priorities of health interventions in the National Health Policy, Strategy or Plans (NHPSPs) of 38 OECD countries and 5 BRICS nations before and after the COVID-19 pandemic: the study reported that 94.4% of the countries analyzed identified prevention efforts, in terms of promoting and protecting public health, as a high-priority health-setting area. Despite this progress, the existing literature still contains knowledge gaps, for example regarding the combination of structural differences between healthcare systems with long-term quantitative analyses of specific expenditure components, such as preventive care, and the analysis of certain health determinants linked to the healthcare context as potential barriers to the implementation of solutions that put into practice the recommendations contained in the NHPSPs of individual countries.

Aiming to bridge knowledge gaps against this background, this study is the first, to our knowledge, to combine a long-term (2004–2023) trend analysis of preventive care expenditure with a cross-country comparative perspective across different healthcare system models in Europe examining whether specific health systems are associated with different time trends in preventive-care expenditure share, thereby contributing to a more comprehensive understanding of how healthcare systems may shape budgetary priority assigned to preventive care.

## Methods

A time trend analysis was performed using secondary data from 22 European OECD countries during a twenty-year period (2004–2023). The countries included in the study were: Austria, Belgium, Czech Republic, Denmark, Estonia, Finland, France, Germany, Hungary, Iceland, Ireland, Italy, Luxembourg, Netherlands, Norway, Poland, Portugal, Slovak Republic, Spain, Sweden, Switzerland and the United Kingdom. These countries and years were chosen based on the availability of data. We obtained official data from the Organization for Economic Co-operation and Development (OECD). The primary dependent variable was the percentage of total current health expenditure allocated to preventive care, calculated from values expressed in Euros, PPP converted, 2020. We interpreted this indicator as a measure of the relative priority assigned to prevention within the budget allocated to health care spending, rather than as a direct measure of absolute preventive-care expenditure, or service volume.

Over time, several approaches have been proposed to classify healthcare systems. We adopted the typology that was presented by Rothgang and Wendt (16) and modified by Böhm (9) because it attempts a deductive construction of healthcare system types and allows for a more precise classification of healthcare systems. According to Böhm, each health care system is identified by three dimensions that are not entirely independent of each other but follow a clear order: the regulation dimension is first, followed by the financing dimension and finally service provision. In every dimension, three actors can play a role: state, societal or private actors (see Additional file 1 for a summary of Böhm's classification) (9). Consequently, healthcare systems in OECD countries can be grouped under five main models. In addition to the time trend analysis, we performed a regression analysis including the three structural dimensions — regulation, financing and provision— according to Böhm's classification as explanatory variables in order to explore their association with the preventive-care expenditure share at the individual country level.

### Statistical analysis

We performed a hierarchical semi-log polynomial mixed-effects regression analysis of annual country-level preventive-care expenditure share, specifying random intercepts and random time slopes to account for unobserved heterogeneity across countries and the repeated-measures (country–year) structure of the panel (17). Observations were considered to years ≥2004, and time was re-parameterized as a re-centered linear index [year_linear = year – (min (year) – 1)] with a quadratic term (year_linear^∧^2) to capture curvature while improving numerical stability. The dependent variable was log-transformed [log (preventive-care expenditure share)] to improve model fit under convex/exponential time patterns and to support interpretation on a multiplicative scale. Fixed effects included year_linear, year_linear^∧^2, and three categorical health system dimensions (regulation, financing, provision), each coded with “Statal” as the reference level, along with interactions between each dimension and both time terms to assess whether health system typologies were associated with different trajectories in the preventive-care expenditure share over time. The random-effects structure included a country-specific random intercept and country-specific random slopes for both year_linear and year_linear^∧^2. Models were estimated by maximum likelihood using lmer from the lme4 package (17).

Because inference in mixed-effects models can be sensitive with a limited number of higher-level units, we quantified uncertainty for fixed-effect parameters using a model-based parametric bootstrap with 1,000 replicates, implemented via bootMer, extracting the fixed effects at each replicate to obtain bootstrap standard errors and normal-approximation 95% confidence intervals and two-sided *p*-values (17, 18). Random-effects variability was summarized using the estimated standard deviations of the country-level random intercept and random slopes, alongside the residual (country–year) standard deviation from the fitted model. To support interpretation of between-group differences, we derived estimated marginal means (overall level differences) and estimated marginal trends (differences in slopes with respect to year_linear) for each health system dimension using the emmeans framework, including global joint tests and pairwise contrasts (19). For visualization and reporting, fitted values and confidence limits computed on the log scale were back-transformed to the original preventive-care expenditure share scale using the exponential function. All analyses were conducted in R version 4.3.1 (2023-06-16) using the major packages lme4 (model estimation and parametric bootstrap), emmeans (marginal means and trends), dplyr/tidyr/purrr (data management), and ggplot2 (visualization) (17, 19–23).

## Results

**Figure 1:**
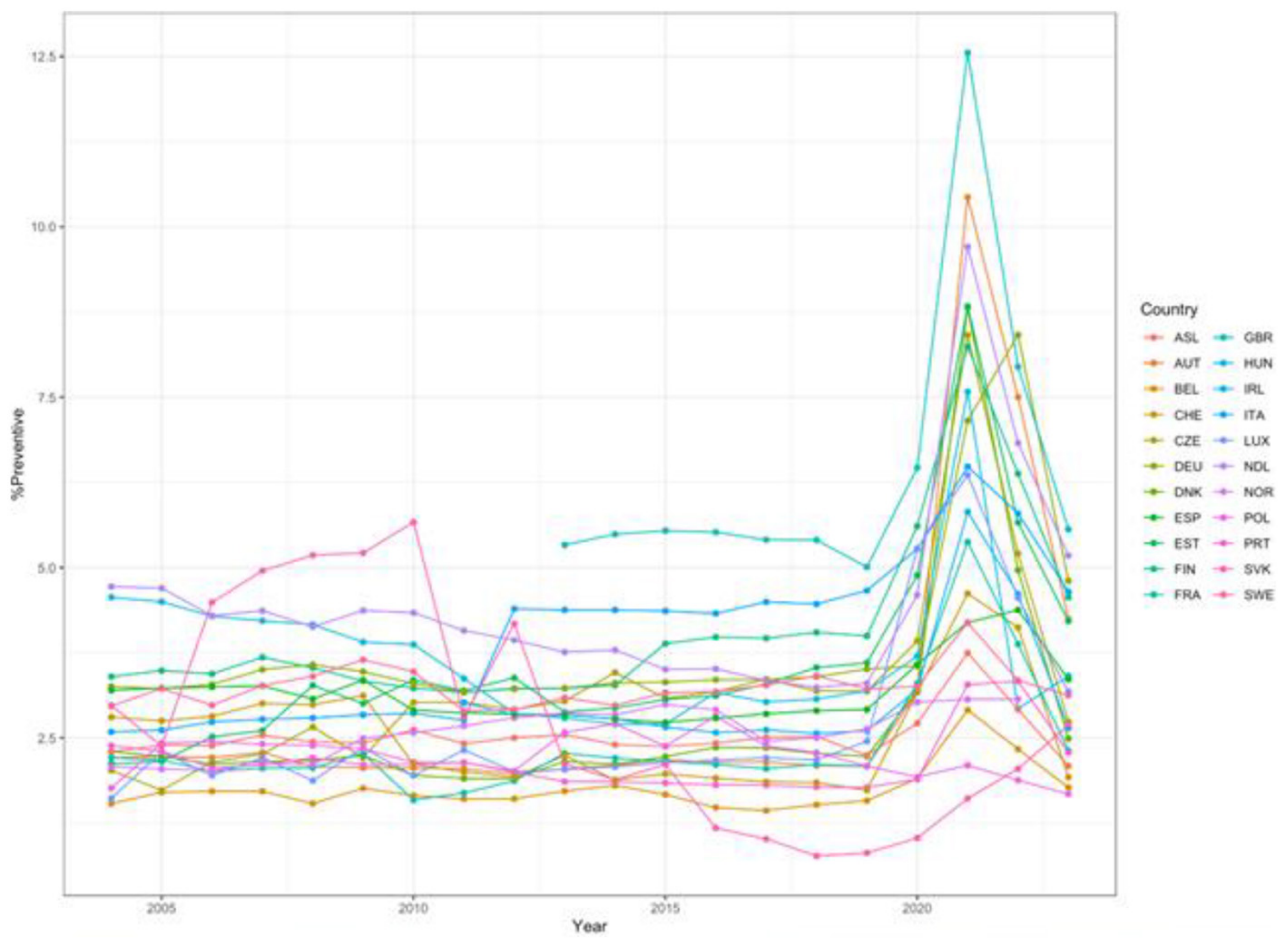
Share of total health expenditure allocated to preventive care, in 22 European countries, 2004–2023. ASL, Iceland; AUT, Austria; BEL, Belgium; CHE, Switzerland; CZE, Czech Republic; DEU, Germany; DNK, Denmark; ESP, Spain; EST, Estonia; FIN, Finland; FRA, France; GBR, United Kingdom; HUN, Hungary; IRL, Ireland; ITA, Italy; LUX, Luxembourg; NDL, Netherlands; NOR, Norway; POL, Poland; PRT, Portugal; SVK, Slovak Republic; SWE, Sweden.

Figure 1 displays the share of total health expenditure allocated to preventive care in 22 European countries examined. Most countries exhibited an increase in the preventive-care expenditure share during 2004–2023, and seven countries (Hungary, Ireland, Iceland, Poland, Portugal, the Slovak Republic, and Switzerland) showed a decrease during the period studied. Figure 1 highlights that there is a wide range of initial and the final preventive-care expenditure share, and shows a heterogeneous trend whereby countries did not maintain the same position in the final ranking compared to the initial one. All countries showed a peak in 2021, during the COVID-19 period.

Table 1 presents the compound annual rate for each country. Over the full observation period, the preventive-care expenditure share increased at a compound annual rate between 0.2 and 3.7% in most countries. Conversely, in seven countries, preventive-care expenditure share decreased at a compound annual rate between −6.3 and −0.2% and, with the exception of Poland, confirmed the decreasing trend already recorded in the period 2004–2019. All countries showed a sharp increase during the COVID-19 period (2019–2021), followed by a reduction in the preventive-care expenditure share in the following 2 years (2022–2023).

**Table 1:** Compound annual rate of the preventive-care expenditure share (%) and types of regulation, financing and provision for each country.

Countries	Regulation		Financing		Provision		Compound annual rate 2004–2019 (%)	Compound annual rate 2019–2021 (%)	Compound annual rate 2021–2023 (%)	Compound annual rate 2004–2023 (%)
	ST (n.18)	SO (n.4)	ST (n.10)	SO (n.12)	ST (n.8)	PR (n.14)				
Austria		✓		✓		✓	−0, 4	12, 9	−36, 2	3, 5
Belgium	✓			✓		✓	0, 2	35, 8	−22, 0	0, 8
Czechia	✓			✓		✓	3, 1	62, 5	−43, 0	1, 6
Denmark	✓		✓		✓		−0, 2	98, 9	−46, 9	0, 4
Estonia	✓			✓		✓	3, 6	56, 6	−30, 9	3, 7
Finland	✓		✓		✓		1, 1	43, 6	−25, 6	1, 6
France	✓			✓		✓	−1, 4	60, 6	−34, 4	0, 2
Germany		✓		✓		✓	0, 6	42, 8	−18, 1	2, 1
Hungary	✓			✓		✓	−2, 4	54, 2	−33, 0	−1, 5
Iceland	✓		✓		✓		−0, 2	29, 3	−25, 3	−0, 5
Ireland	✓		✓			✓	−1, 8	49, 4	−32, 6	−6, 3^a^
Italy	✓		✓			✓	4, 0	17, 9	−15, 3	3, 1
Luxembourg		✓		✓		✓	2, 8	61, 1	−29, 3	3, 6
Netherlands	✓			✓		✓	−2, 4	71, 5	−27, 0	0, 5
Norway	✓		✓		✓		1, 6	8, 0	NA^b^	2, 2^b^
Poland	✓			✓		✓	1, 1	0, 3	−10, 6	−0, 3
Portugal	✓		✓		✓		−2, 0	36, 0	−16, 5	−0, 2
Slovak Republic	✓			✓		✓	−8, 3	40, 9	−29, 2	−0, 5
Spain	✓		✓		✓		−0, 6	19, 9	−10, 5	0, 3
Sweden	✓		✓		✓		0, 5	14, 2	−13, 7	0, 3
Switzerland		✓		✓		✓	−3, 2	63, 3	−35, 5	−2
United Kingdom	✓		✓		✓		−1, 0	58, 3	−33, 4	0, 4^c^

Ticks note the types of Regulation, Financing and Provision for each country. ST, Statal; SO, Societal; PR, Private; N/A, not available.

^a^data not available 2004–2010.

^b^data not available 2023.

^c^data not available 2004–2012.

Table 1 displays the classification of each healthcare system according to the three dimensions too.

Eight countries had statal regulation, financing and provision, 8 had statal regulation, societal financing, private provision; four countries had societal regulation and financing with private provision; two had statal regulation and financing with private provision.

Table 2 displays the results of regression analysis. The trend in the preventive-care expenditure share did not differ in two of the three dimensions under study: financing and provision. In contrast, in countries with societal regulation, the curvilinear trend per year was more pronounced than in countries with statal regulation (*b* = 0.0035; 95% CI = 0.0013, 0.0057), however the difference observed over the entire observation period is not statistically significant.

**Table 2:** Results from hierarchical semi-log polynomial mixed-effects regression models of the preventive-care expenditure share in 22 European countries, 2004–2023.

Variable	Regression coefficient	Bootstrap standard error	*p*-value	Normal-based 95% CI	
(Intercept)	1.055	0.0989	0	0.8612	1.249
Year	−0.0216	0.0154	0.1596	−0.0517	0.0085
Year^2^	0.0019	< 0,001	0.0044	< 0,001	0.0032
Societal regulation (ref. statal)	0.0383	0.1596	0.8103	−0.2745	0.3512
Societal financing (ref. statal)	0.2864	0.2765	0.3002	−0.2555	0.8283
Private provision (ref. statal)	−0.2501	0.2699	0.3542	−0.7791	0.2789
Societal regulation x year	−0.0509	0.0261	0.051	−0.102	< 0,001
Societal regulation x year^2^	0.0035	0.0011	0.0021	0.0013	0.0057
Societal financing x year	−0.0688	0.042	0.1014	−0.151	0.0135
Societal financing x year^2^	0.0018	0.0017	0.2853	−0.0015	0.0052
Private provision x year	0.0571	0.0409	0.1626	−0.0231	0.1373
Private provision x year^2^	−0.0019	0.0017	0.2572	−0.0052	0.0014

95% CI, 95% confidence interval; SD, standard deviation.

The quadratic term, year^2^, indicates the presence of a curvilinear (or nonlinear, U-shaped) trend over time. The interaction terms (marked with the sign “ × ”) indicate how much year and year^2^ are different for different health care system types. So, year and year^2^ represent the linear and quadratic slope when regulation, financing and provision are issued by public actors, i.e., the reference category for each dimension.

**Figure 2:**
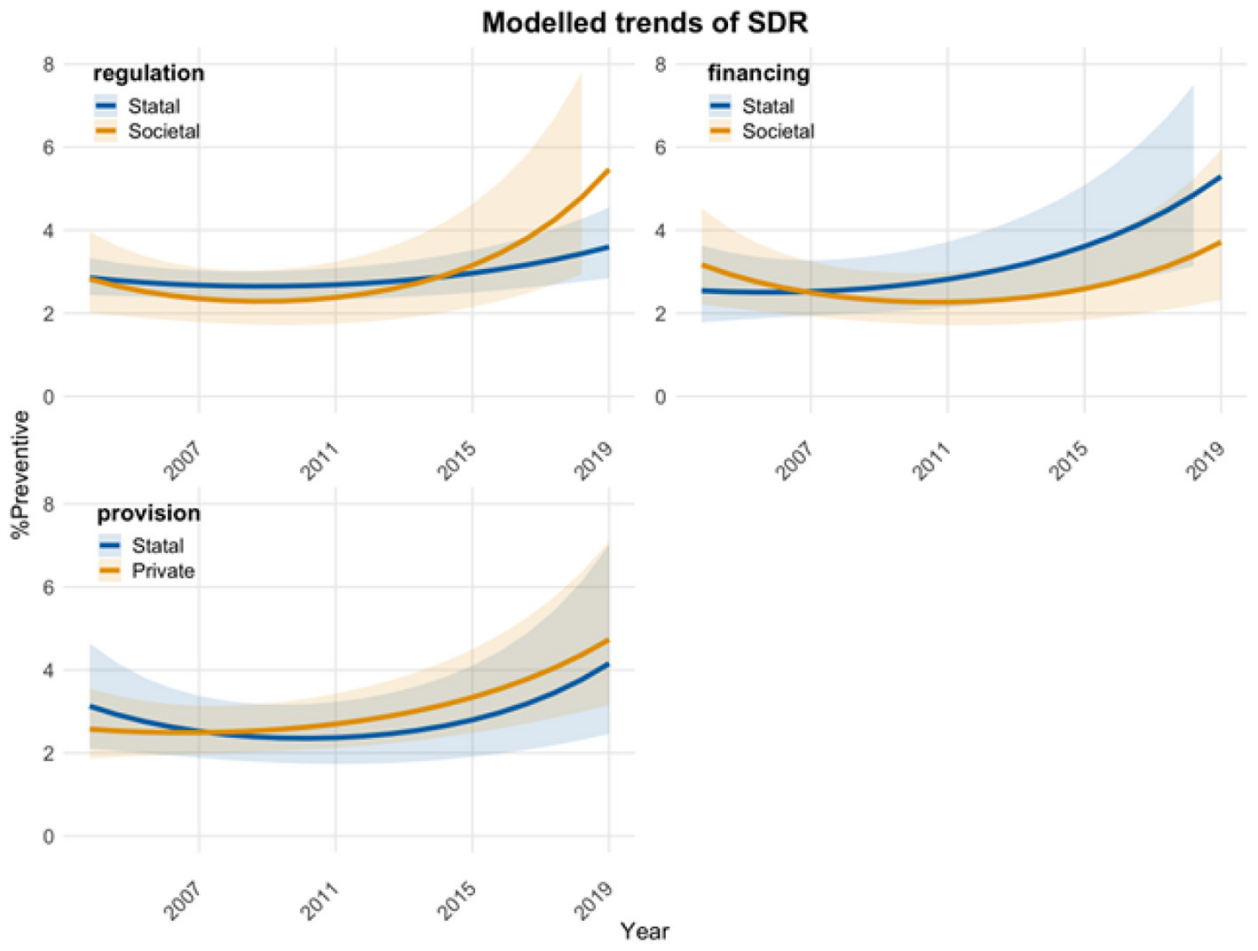
Modeled trends in the preventive-care expenditure share, stratified by dimensions, 2004–2019.

Results of regression analysis are illustrated in Figure 2 where % preventive expenditures are stratified by dimension and plotted for each year. Countries with societal regulation showed a more positive nonlinear trajectory in the preventive-care expenditure share, although this pattern should be interpreted cautiously.

## Discussion

This study analyzed time trends in the share of health expenditure allocated to preventive care and explored whether there was an association between trends and a given healthcare system model. To this end, the analysis was based on data from 22 European countries, using the preventive-care expenditure share as the primary outcome and summarizing country-specific changes through compound annual growth rates.

In 15 out of 22 countries, an increase in the preventive-care expenditure share has been observed over the considered period (2004–2023), with a compound annual growth rate peaking at 3.7%. In the remaining seven countries, however, there was a decrease in the relative share of health expenditure allocated to preventive care, with a compound annual growth rate reaching a low of −6.3%.

Despite increases in preventive-care expenditure share in some countries, the share remains marginal compared to total healthcare spending, amplifying the findings of Gmeinder et al., who reported the same problem in Europe for the period from 2005 to 2015 (24).

This pattern is consistent with the broader literature describing prevention as an “easy target” for budgetary restraint because its benefits are only visible in the long term, and there is usually no professional or commercial pressure in its favor. For this reason, prevention is a “quiet” policy that does not enjoy the same public support as policies that support interventions in the curative sphere, which cover immediate needs shared by most citizens (4, 10). This modus operandi seems to be a common trend in all countries, regardless of political orientation, probably driven by the need for immediate returns in economic terms and consensus (4, 25).

Despite short-term political gains, several authors argue that cutting spending on prevention is a “false economy” (10, 26, 27). A systematic review attempted to quantify the impact of public health interventions delivered in high-income countries, defined as “the science and art of promoting and protecting health and wellbeing, preventing ill-health and prolonging life through the organized efforts of society”: the median return on investment was 14.3–1, and the median cost-benefit ratio was 8.3 (26). Under-prioritizing preventive care to achieve short-term savings could not only reduce the benefits in terms of improving quality of life (28–30), but also have negative consequences for society in the long term, increasing pressure on healthcare services and worsening health inequalities (10, 27). In fact, it should be considered that primary or secondary prevention produces significant positive externalities: when a sufficient percentage of the population is vaccinated, the circulation of the pathogen is reduced, protecting the unvaccinated (herd immunity) as well; early diagnosis of a disease not only saves the life of the individual (private benefit), but also reduces social and health costs for the entire community, improving overall public health (4). Furthermore, a major problem with preventive care is the very high asymmetric information: an ordinary citizen/patient is unable to get full information about his/her needs, his/her health risks, as well as about the resulting probability of health care needs and the expenditures which will have to be supported to a health care he will consume (31).

Regarding the temporal trend, our study also identified a peak in the preventive-care expenditure share in 2020 and 2021. The sharp contraction in this share in 2022–2023, shared by all countries, highlights the exceptional nature of the pandemic peak, linked to emergency measures such as vaccination campaigns, testing and tracking for the management of COVID-19 (24, 32). The rapid return of the expenditure share toward pre-pandemic levels, immediately after the pandemic, suggests that healthcare systems did not translate the pandemic-related increase in the preventive-care expenditure share into a sustained re-prioritization of preventive care within health budgets (4, 5). This phenomenon had already been observed during the H1N1 pandemic in 2009, where the need to manage the so-called “swine flu” caused spikes in prevention spending, followed by immediate reductions in subsequent years (24).

The results of the regression analyses show no significant association between the actor responsible for each domain characterizing the specific healthcare system and the relative share of health expenditure allocated to preventive care. The only exception is the dimension of regulation (societal vs. statal): according to our results, this was the only dimension showing evidence of a differential nonlinear trajectory, yet with an unexpected dynamic. A widespread theory in the literature states that National Health Service-type systems, i.e., state-regulated systems, tend to maintain relative priority for preventive care (4). On the contrary, our results show that systems in which regulation is dominated by societal actors tended to report a faster increase in the preventive-care expenditure share, albeit slight. The interpretation of this result is very complex; the explanations proposed below cannot be inferred from our results and should be viewed with caution. The faster rise in the preventive-care expenditure share in Societal Health Insurance (SHI) model could be interpreted considering different approaches to funding and implementing preventive care in comparison with state-regulated systems: the societal regulation system is a mandatory, contribution-based system providing health risk cover through exhaustive benefits packages, and consumers can choose among multiple sickness funds. SHI funds, through self-government, determine their own preventive offers within a legal framework; they often use prevention programmes to compete for members and may use bonuses to incentivise individual participation, which can increase the focus on preventive services (33, 34). Another hypothetical explanation could be the trend toward hybridisation of healthcare systems and the crumbling of old institutional logic (35). However, looking at Figure 2, we can see how this surge in societal regulation systems is almost concurrent with the years of the pandemic. It cannot be ruled out that the above mentioned difference is influenced by the impact of the COVID-19 pandemic and that the latter has disrupted the “classic” dynamics described above.

### Strengths and limitations

Although the findings should be interpreted with caution, this study has several strengths: we updated cross-country evidence on the preventive-care expenditure share; we estimated the slope rate as well. The slope of this share provides complementary information on how the relative prioritization of preventive care evolved over time, because the slope rate is less susceptible to confounding factors such as baseline health risk and the socio-epidemiological characteristics of the population. To our knowledge, this is the first study that examines hypothetical associations between specific types of health systems and different patterns in the relative allocation of health expenditure to preventive care, according to Bohm's classification for health systems. Regression analyses of each of the three dimensions - regulation, financing, provision - on the percentage of preventive expenditure may represent independent sources of action and interest in health policy, although these dimensions are structurally related and should not be interpreted as independent causal effects. Furthermore, although it was not possible to carry out the analysis on all OECD countries, this study conducts a comparative analysis on a large sample of European countries, allowing the phenomenon to be explored in different health and economic contexts.

A limitation of this study is that this analysis has been conducted at the aggregate preventive-care expenditure category level, and has not disaggregated expenditures by specific types (primary, secondary, tertiary); this potentially could conceal variations among countries on the content and range of services offered to citizens/patients. Consequently, analysis at the national level must be conducted to assist policymakers in making better-informed decisions about preventive care.

## Conclusions

In conclusion, for the countries analyzed, our time trend analysis shows a rather heterogeneous annual rate of change in the share of health expenditure allocated to preventive care in the considered period, yet a generally limited relative prioritization of preventive care can be highlighted.

This study found limited evidence of an association between the type of healthcare system and the share of expenditure allocated to prevention activities in the countries analyzed. With the aim of contributing to the development of healthcare policies, our study provides a useful starting point for future research designed to investigate the causal relationship between these two dimensions: the results show that the relative budgetary prioritization of preventive care in the considered period was not associated with the organizational structure of the healthcare system; on the other hand, this relative prioritization may also reflect contextual factors, such as internal economic and social needs and political decisions. This assumption strengthens the idea that there is a strong social and political responsibility to maintain adequate and prioritization of preventive care, alongside adequate absolute investment over time, regardless of major events affecting society (e.g., the COVID-19 pandemic) and the existing healthcare system model.

Future studies should explore the role of political and contextual determinants, focusing on the most effective communication methods for guiding healthcare policy decisions.

## Data Availability

The original contributions presented in the study are included in the article/supplementary material, further inquiries can be directed to the corresponding author.
